# Negative outcomes evoke cyclic irrational decisions in Rock, Paper, Scissors

**DOI:** 10.1038/srep20479

**Published:** 2016-02-04

**Authors:** Benjamin James Dyson, Jonathan Michael Paul Wilbiks, Raj Sandhu, Georgios Papanicolaou, Jaimie Lintag

**Affiliations:** 1 University of Sussex, Falmer, BN1 9QH, UK; 2 Ryerson University, 350 Victoria St, Toronto, ON M5B 2K3, Canada

## Abstract

Rock, Paper, Scissors (RPS) represents a unique gaming space in which the predictions of human rational decision-making can be compared with actual performance. Playing a computerized opponent adopting a mixed-strategy equilibrium, participants revealed a non-significant tendency to over-select Rock. Further violations of rational decision-making were observed using an inter-trial analysis where participants were more likely to switch their item selection at trial *n* + *1* following a loss or draw at trial *n*, revealing the strategic vulnerability of individuals following the experience of negative rather than positive outcome. Unique switch strategies related to each of these trial *n* outcomes were also identified: after losing participants were more likely to ‘downgrade’ their item (e.g., Rock followed by Scissors) but after drawing participants were more likely to ‘upgrade’ their item (e.g., Rock followed by Paper). Further repetition analysis revealed that participants were more likely to continue their specific cyclic item change strategy into trial *n* + *2*. The data reveal the strategic vulnerability of individuals following the experience of negative rather than positive outcome, the tensions between behavioural and cognitive influences on decision making, and underline the dangers of increased behavioural predictability in other recursive, non-cooperative environments such as economics and politics.

The dynamic system represented by game Rock, Paper, Scissors (RPS) is both a physical reality in the animal world (specifically amongst three species of side-blotched lizards^1^) and serves as an important paradigm for assessing the degree of rational decision making inherent within non-cooperative environments across species (e.g.^2^,^3^). In a typical RPS game, participants reveal a three-alternative choice at the same time: Rock, Paper or Scissors. The winner (and loser) is calculated by the rule Rock wins over Scissors (the Scissors are ‘blunted’), Paper wins over Rock (the Rock is ‘covered’) and Scissors wins over Paper (the Paper is ‘cut’). The specific relationships between elements (*non-transitive dominance relations*^4^) dictate a crucial aspect of the game space, in that there is no singular strategy that guarantees success (or *evolutionarily stable strategy*; Maynard-Smith & Price, 1973, cited in^3^). As such, when it is played in a potentially infinitely recursive manner, the various responses may enjoy periods of temporary dominance and the lack of a definitive strategy becomes particularly apparent when children can often serve as formidable opponents to adults^5^.

One aspect of the game that has received particular interest results from the unique Nash equilibrium of RPS^5^, where “an equilibrium point is a pair of strategies that are best replies to each other, a best reply being a strategy that maximizes a player’s payoff, given the strategy chosen by the other player” (3, p. 140). During RPS, players should adopt a mixed-strategy equilibrium wherein multiple items are played stochastically^2^. In other words, each of the three items should be played with random distribution but equal probability (33.33%). The disadvantages of not following this strategy are made clear by^6^, who show that if a computer opponent plays one item more often than another (e.g., Rock) then human participants will play the appropriate counter-item with increased frequency (e.g., Paper). Under this scheme, the computer opponent would be said to be playing a strategy that could be *dominated* and serves as an example of irrational (not to mention evolutionarily unsound^3^) decision making. Therefore, precisely constructed game environments such as RPS supply researchers with a baseline against which deviations from rational decision-making may be observed and predicted. As^7^ (p. 55) state: “equilibrium strategies specified by game theory provide a precise yardstick to quantify how human behaviors deviate from the normative predictions for rational players. One can therefore try to identify factors responsible for the discrepancy between the normative predictions and observed behaviors”.

The first indication of irrationality in RPS comes from the observation that specific items in game systems may be naturally favoured. Decision making can be influenced by saliency^8^, where *primary salience* refers to selecting responses that more readily come to mind (c.f., *availability heuristic*^9^) and *secondary salience* refers to adopting a strategy whereby one assume the opponent is operating on the basis of primary salience. Evidence of primary salience in RPS comes from^10^ who reported that across 300 rounds, participants selected Rock 35.66%, Paper 32.12% and Scissors 32.23% of the time, and a similar bias for Rock was also reported by^4^ with Rock 36%, Paper 33% and Scissors 32%. Therefore, there might be a simple influence at the item level that draws individuals away from adhering to a mixed-strategy equilibrium and, ultimately, rationality^3^. Similarly, secondary salience might also contribute to RPS performance according to the observations of^6^: my opponent is predominantly playing Rock so I will predominantly play Paper. However, such strategies cannot be evolutionarily stable due to the recursive nature of the game: eventually an opponent may eventually adjust *their* strategy according to *your* overplaying of Paper.

Ref. 4 also showed that participants implement an apparently successful rule-based strategy of *if I win then I stay with my current item, if I lose then I shift to a new item* (‘win-stay, lose-shift’; see also^7^, for a similar strategy in monkeys). In contrast to the memorial and cognitive demands of the mixed-strategy equilibrium (frequency counting three-alternative choices across hundreds of rounds to ensure that each item is played 33.33% of the time), tendencies to adopt rules like ‘win-stay, lose-shift’ become “psychologically plausible for human subjects with bounded rationality” 4(p. 5). Principles such as Thorndike’s *Law of Effect* (Thorndike, 1911, cited in^11^) and the *matching law* (Hernstein, 1961, cited in^6^), where the proportion of response matches the degree of reinforcement, seem to fit well with an account where participants maintain their current course of action in the light of success (‘win-stay’) but change their current course of action in the light of failure (‘lose-shift’; see also^12^, for a similar *criterion of progress* in the context of problem solving). Although necessary on the basis of limited human cognition, predictable consequences as a function of *winning* (reinforcement; *stay*) or *losing* (punishment; *switch*) also run the risk of being dominated. Furthermore, neural activity associated with reinforcement and punishment in the context of RPS trial outcome are found throughout the cortex and to a much larger degree than previously thought, with additional specific areas distinguishing between *win* and *loss* (accumbens, caudial ACC and transverse temporal region) and also between *stay* and *switch* (medial frontal cortex and caudate^13^). The cortex-encompassing activity associated with trial outcome has the required distributed nature to impact on numerous cognitive processes.

However, switch heuristics such as *win-stay lose-shift* remain underspecified at the level of item selection and two additional categories of response change suggest themselves (after^4^). First, participants may choose to *downgrade* their response across trials, defined as selecting the item in trial *n* + *1* that would have been beaten by their item at trial *n* (e.g., Rock followed by Scissors; also ‘descending’^14^ or ‘left-shift’^15^). Alternatively, participants may choose to *upgrade* their response across trials, defined as selecting the item in trial *n* + *1* that would have beaten their item at trial *n* (e.g., Rock followed by Paper; also ‘ascending’^14^ or ‘right-shift’^15^). Due to the cyclical nature of the relationships between items (see Fig. 1a) it would be possible to repeat the strategy of upgrading or downgrading across multiple consecutive trials. Of additional importance are the cognitive implications of *draw* trials, which are also currently underspecified. It may seem reasonable to assume that *draw* trials should be less arousing than *win* or *loss* trials and so have less on an impact on subsequent performance, but a recent study provided no evidence that this was the case^13^.

To further investigate the heuristics underlying RPS performance, human participants played 225 rounds of RPS with a computer opponent operating according to the mixed-strategy equilibrium. Human participants were not made aware of the specific strategy of the computer at the time of testing, given that the absence of instruction is important in understanding real-world decision making where information also tends to be incomplete^6^. Response proportions across consecutive trials were examined in terms of the item selected at trial *n* (Rock, Paper, Scissors), the outcome at trial *n* (*win, lose, draw*) and the strategy subsequently deployed at trial *n* + *1* relative to *n* (*stay, upgrade, downgrade*). The distribution of responses across three trials were also examined in terms of the strategy deployed between trial *n* and *n* + *1* (after^4^) and between trial *n* + *1* and *n* + *2* (*stay, upgrade, downgrade*). Any interactions revealed between these levels would undermine the view of human decision making as rational and, importantly, define the item-based and outcome-based conditions under which such violations could be predicted.

## Results

### Trial n item selection and outcome

Concerns regarding the violation of normality in the data set were addressed by carrying out arc-sine transformations on the proportion data, according to the suggestion of^16^. The formula 2*(ASIN(SQRT(*x*)) was used when proportion error rates were greater than zero and *x* represents the proportion, and, 2*(ASIN(SQRT(1/2 *y*))) was used when proportion error rates were equal to zero and *y* represented the number of possible observations within a cell. The main effects and interactions of the transformed data were equivalent to those generated by the original proportion data. To aid the interpretation of the descriptive statistics, we have retained the primary analysis of proportion data here although both the results from the transformed data and raw proportion data can be found in Supplementary Materials.

Using separate one-way repeated measures ANOVAs with the factors of item selection at trial *n* (rock, paper, scissors) and outcome at trial *n* (*win, lose, draw*), respectively, we confirm that participants did not significantly differ in terms of their item selection at trial *n* [F(2, 60) = 1.64, MSE = 368.53, *p* = 0.203, η_p_^2^ = 0.052] or the outcome at trial *n* [F(2, 60) = 1.21, MSE = 94.85, *p* = 0.306, η_p_^2^ = 0.039]. In terms of item selection at trial *n*, there was a non-significant tendency for participants to play Rock (80.10 trials; 35.60%) slightly more often than Paper (72.55 trials; 32.24%) or Scissors (72.35 trials; 32.16%). In terms of outcome at trial *n*, participants had a non-significant tendency to win (77.10 trials; 34.27%) slightly more than they drew (73.32 trials; 32.59%) or lost (74.58 trials; 33.15%).

### First-order repetition effects

**Figure 1:**
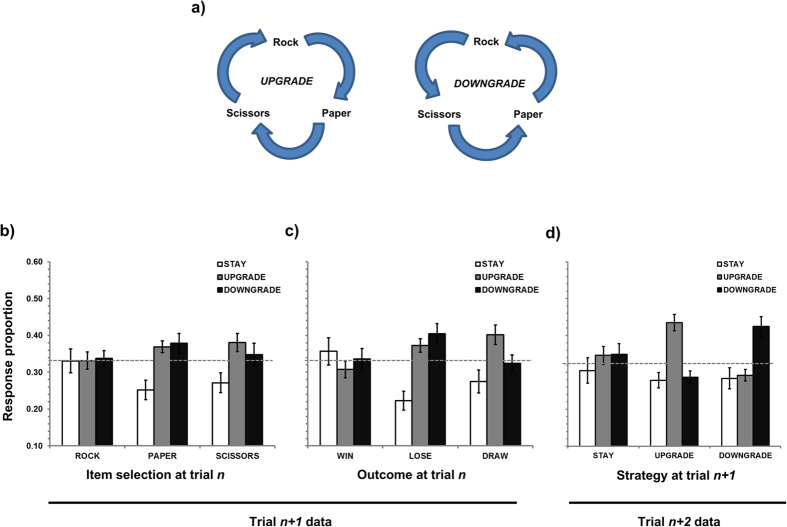
(**a**) Schematic showing the cyclical nature of upgrading or downgrading responses in Rock, Paper, Scissors, (**b**) Graph showing strategy at trial *n* + *1* as a function of item selection at trial *n*, (**c**) Graph showing strategy at trial *n* + *1* as a function of outcome of trial *n*, (**d**) Graph showing the strategy adopted between trial *n* + *1* and *n* + *2* as a function of the strategy adopted between trial *n* and *n* + *1.*

Proportion data using 222 trials (the first trial in each block had no previous history) were analysed according to a three-way repeated measures ANOVA using the factors of item selection at trial *n* (rock, paper, scissors), outcome at trial *n* (*win, lose, draw*) and strategy at trial *n* + *1* (*stay, downgrade, upgrade*). Since the strategy at trial *n* + *1* data were calculated as proportion of both item selection at trial *n* and outcome at trial *n*, the analysis of these trial *n* terms (and the resultant interaction) was meaningless (*p* = 1). The main effect of strategy was not significant [F(2, 60) = 2.70, MSE = 0.183, *p* = 0.075, η_p_^2^ = 0.083], nor was the three-way interaction between item x outcome x strategy [F(8, 240) = 1.59, MSE = 0.012, *p* = 0.127, η_p_^2^ = 0.050]. However, significant two-way interactions between item x strategy [F(4, 120) = 2.71, MSE = 0.048, *p* = 0.033, η_p_^2^ = 0.083] and outcome x strategy [F(4, 120) = 6.71, MSE = 0.061, *p* < 0.001, η_p_^2^ = 0.183] were observed. Figure 1b shows strategy at trial *n* + *1* as a function of item selection at trial *n*, which was decomposed according to Tukey’s HSD test (*p* < 0.05). Playing Rock at trial *n* did not seem to impact on strategy at trial *n* + *1*, following Paper participants were more likely to switch (either *upgrading* or d*owngrading*; numerically more likely for *downgrading*) than to *stay*, and after Scissors there was a preference for switching (specifically *upgrading*) relative to *staying*.

Figure 1c shows strategy at trial *n* + *1* as a function of outcome at trial *n*, which was again decomposed according to Tukey’s HSD test (*p* < 0.05). No significant differences in trial *n* + *1* strategy were observed following a *win* although numerically the data were in favour of *staying.* In contrast, a *loss* more likely prompted item *switching* than *staying* (numerically in favour of item downgrading) and following a *draw*, participants were inclined to *switch* and specifically *upgrade* their item relative to *staying*.

### Second-order repetition effects

Proportion data using 219 trials (the first 2 trials in each block had no immediate second-order history) were analysed according to a two-way repeated measures ANOVAs using the factors of strategy at trial *n* + *1* (*stay, downgrade, upgrade*) and strategy at trial *n* + *2* (*stay, downgrade, upgrade*). Proportion data was calculated from strategy at trial *n* + *1* rendering that main effect meaningless (*p = *1). An interaction between strategy at trial *n* + *1* and *n* + *2* was revealed [F(4, 120) = 13.12, MSE = 0.012, *p* < 0.001, η_p_^2^ = 0.304] in the absence of a main effect of strategy at trial *n* + *2* [F(2, 60) = 2.19, MSE = 0.063, *p* = 0.121, η_p_^2^ = 0.068]. As shown in Fig. 1d and decomposed using Tukey’s HSD test (*p* < 0.05), staying with item selection between trial *n* and *n* + *1* failed to impact on the strategy adopted between trial *n* + *1* and *n* + *2*. However, participants who upgraded were more likely to continue upgrading and participants who downgraded were more likely to continue downgrading.

## Discussion

Human participants played RPS against a computer opponent with a mixed-strategy equilibrium^2^,^5^. Therefore, any deviation from the equal play of each of the three items (Rock, Paper, Scissors) and any predictable change in strategy (*stay, upgrade, downgrade*) as a function of the outcome of the previous trial (*win, lose, draw*) would place the human participant in a potentially exploitable position. The data provide only limited evidence of *primary salience*^8^ in the context of RPS, but nevertheless replicate the observations of^4^,^10^,^14^ in terms of participants’ tendency to overplay Rock. In terms of the origin of this effect, anecdotally some participants (and indeed some researchers) intuitively believed that Rock was somehow ‘better’ or ‘stronger’ that its Paper and Scissors counterparts, despite the fact that this assumption exists nowhere in the structure of the game. Future research examining both personal reinforcement history and connotative meaning^17^ of individual items in RPS might help to account for the subjective assumptions associated with favored items. If there is a tendency for Rock to be overplayed, then slightly overplaying Paper might be a sensible option against any future opponent^6^. However, since RPS has no evolutionarily stable strategy if the game is played iteratively^5^ the success of this strategy can only be short lived. Interestingly, human participants’ approximation of a mixed-strategy equilibrium in the light of their opponent playing the same strategy runs contrary to the performance of rhesus monkeys. In a simpler matching-pennies games^7^, monkeys adopted a mixed-strategy equilibrium only when the computer’s algorithm adjusted its strategy according to the frequency distribution of the monkey (*secondary salience*^8^). When the computer played according to a mixed-strategy equilibrium, monkeys deviated from equilibrium. Such decision-making differences between animals and humans are common and often reflect superior performance in the former (e.g.^18^ for pigeons’ enhanced sensitivity to base-rate information^19^ for pigeons’ improved performance at the Monty Hall problem). Given that decision-making is also partly determined by whether participants believe their opponent to be a human or machine^20^,^21^, perceived agency continues to be a critical avenue for future human and animal research.

In terms of the inter-trial analysis, the data both replicate and give increased specificity to the ‘win-stay, lose-shift’ strategy. Although consistent with behaviourist principles, the implementation of such a potentially dominated heuristic in recursive, non-cooperative environments places the user in an evolutionarily unsound position. As seen in Fig. 1c, there was a non-significant tendency to *stay* with the previous item selection following a *win* and to *switch* (more specifically, *downgrade*) item selection following a *loss*^15^. Such data are consistent with classic behaviourist principles where reinforced responses are more likely to be used again (‘*win-stay*’) and non-reinforced responses are less likely to be used again (‘*lose-shift*’). However, despite the RPS game space assigning equivalent weight to winning or losing (relative to drawing), gains and losses of equivalent magnitude do not necessarily have the same subjective value^22^. That is, the magnitude of difference between the three potential strategies was larger following *loss* than *win* trials. Therefore, consistent with the initial ideas of^22^, a *loss* impacts on rational decision making to a greater degree than a *win* and individuals become more predictable (and therefore more vulnerable) in terms of their strategy following a loss trial. *Drawing* with an opponent leads to the same overall strategic consequence than *losing* (i.e., *switch*) and so in this respect both *losing* and *drawing* may index a lack of reinforcement and an unsuccessful outcome (c.f.^13^). However, the subsequent nature of the switch is qualitatively different^23^: *losing* promotes *downgrading* whereas *drawing* promotes *upgrading*. Furthermore, those tendencies to *upgrade* or *downgrade* were perpetuated across three consecutive trials (Fig. 1d) lend credence to the idea that there also can be repetition in the nature of change, and that these different forms of switch may have quite different cognitive and emotional consequences. This signposts future work examining how specific emotions may determine movement away from rational decision making (see^24^ for a review), and the trial-to-trial bases on which these occur (see^25^ for an exploration of regret aversion on consecutive decision making).

Of final interest are the implications of the lack of a significant three-way interaction between item selection at trial *n*, outcome at trial *n* and strategy at trial *n* + *1* during first-order repetition effect analysis. Comparing the data across Fig. 1c,d, one can see that both item selection at trial *n* and outcome at trial *n* had very similar impacts on strategy at trial *n* + *1*. To summarize an additive model of trial *n* + *1* strategy that combines item selection and outcome at trial *n*, it is possible to select the item at trial *n* and outcome at trial *n* that produce the largest proportion of responses for each strategy at trial *n* + *1. Stay* responses were associated with selecting Rock relative to Paper or Scissors and *winning* relative to *losing* or *drawing, upgrade* responses were associated with selecting Scissors and *drawing*, whereas *downgrade* responses were associated with picking Paper and *losing.* It is critical to note that in all 3 cases, item selection for the participant at trial *n* + *1* reliably predicts *Rock:* participant *stay* with Rock at trial *n* + *1* following a *win*, Paper is *downgraded* (to Rock) following a *loss*, and Scissors are *upgraded* (also to Rock) following a *draw*. This final observation reveals a much more surprising (and actually much simpler) rule. Unpacking all three contingencies point to the likelihood that *if the computer played Scissors on trial n the participant would be more likely to play Rock on trial n* + *1, irrespective of the item selection and outcome at trial n*. This might explain the non-significant tendency for participants to play Rock overall.

It is worth noting that, although consistent with previous literature, some of observed effects are small, and there may be at least two reasons why this may be so. First, in contrast to other RPS investigations where individuals played a number of opponents^4^,^10^, here each participant played only one computerized opponent who acted according to the Nash equilibrium. It has been suggested that in a two-player RPS game, conditional probabilities should only show minor deviation from 33%^26^. Furthermore, the use of a computerized opponent who failed to take advantage of any player’s vulnerability may have also contributed to a fairly conservative test of irrational decision making. We are currently building various computerized versions of RPS strategy that take into account the player’s actions to varying degrees and we expect this will accentuate the deviations observed in the current data. Second, there was no additional consequence to winning, losing or drawing, other than the immediate feedback presented on screen. We are sensitive to the fact that the financial conversion of performance may well implicate different levels of activity at specific neural sites (such as the nucleus accumbens) from those associated with simple cognitive feedback [e.g.^27^]. We expect to see greater differentiation between *win* and *loss* trials when these are associated with a point and/or monetary system.

The enduring appeal of RPS is partly due to the failure to reveal any optimal strategy when the game is played recursively. Indeed, any collective strategies revealed here (e.g., overplay of Rock, ‘win-stay, lose-shift’, recursive upgrading and downgrading) could be used by the most savvy RPS player in the development of suitable counter-strategies. As such, the RPS framework continues to evolve but remains stable in providing critical insights how we deviate from rational decision making. The present study identifies two negative trial outcomes (losing and drawing) that make irrational decisions more likely, with each outcome leading to different behavioural change (*downgrading* and *upgrading*). Such strategies are also more likely to be perpetuated across multiple trial outcomes, assumedly as a result of the constraints of human information processing. Revealed through the game space of RPS, the tendency to initiate any form of potentially dominated strategy as a result of the micro-emotional responses associated with negative outcome may have more serious implications in other continuous non-cooperative interactions such as economics and politics.

## Method

### Participants

31 undergraduate students (26 female) participated in the study; mean age was 20.23 years (SD = 5.66; one participant did not provide their age) and 27 were right-handed. 2 additional participants were excluded for recording responses at each trial using paper and pencil, assumedly in a systematic attempt to work out the computer’s strategy. Relative to the other participants who used internal working memory only, we felt this did not represent the same test of bounded rationality. The study was approved for testing by the Research Ethics Board of Ryerson University and the study was carried out in accordance with the approved guidelines. Informed consent was obtained from all participants, and course credit was offered for participation.

### Stimuli and apparatus

Visual representations of a hand making Rock, Paper and Scissors signs sourced from the internet were displayed at a visual angle of approximately 4.5° × 4.5° with participants sat approximately 57 cms away from the screen. All text was white and presented on black background. The presentation of stimuli was controlled by *PsyScope* (Cohen, MacWhinney, Flatt & Provost, 1993) and responses were recorded using a *PsyScope* Button Box. The *PsyScope* script is available upon request.

### Design and procedure

Following a set of specific on-screen instructions (see Supplementary Materials), participants completed 225 trials of RPS separated across 3 blocks of 75 trials. In each block, the computer played Rock, Paper and Scissors 25 times in a random order (mixed-strategy equilibrium^2^,^3^,^7^). At each trial, participants were prompted to press one of three buttons corresponding to Rock, Paper and Scissors, following the signal ‘GO!’ At the time of pressing, the computer displayed their selection on the left, the participant’s selection on the right for 1000 ms. After a response checking period of 100 ms, feedback was provided for a further 1000 ms as to whether the participant won, lost or drew the trial. The next trial started following a 250 ms blank screen. Participants were verbally informed that the computer would play in a certain way (which would be revealed after the experiment) and that they were to try to beat the computer across the course of the game. Prior to RPS completion, participants completed a short mental exercise which was designed to manipulate the perceived agency of the computer (see Supplementary Materials). Since this manipulation appeared unsuccessful, data were collapsed across agency.

## Additional Information

**How to cite this article**: Dyson, B. J. *et al*. Negative outcomes evoke cyclic irrational decisions in Rock, Paper, Scissors. *Sci. Rep.* **6**, 20479; doi: 10.1038/srep20479 (2016).

## Supplementary Material

Supplementary Information

Supplementary Information
