# Supplementary material for: Negative outcomes evoke cyclic irrational decisions in Rock, Paper, Scissors
Source: Sci Rep. 2016 Feb 4;6:20479. doi: 10.1038/srep20479 (PMC4740902; doi:10.1038/srep20479)
Supplement: Supplementary Information [file srep20479-s1.pdf]

Negative outcomes evoke cyclic irrational decisions in Rock, Paper, Scissors

Benjamin James Dyson, Jonathan Michael Paul Wilbiks, Raj Sandhu, Georgios Papanicolaou,

Jaimie Lintag

On-screen instructions presented at each block

You are invited to play Rock, Paper, Scissors over 3 blocks of 75 trials.
At each trial, decide what you will select.
After the prompt GO!, press

RED for ROCK
YELLOW for PAPER
GREEN for SCISSORS

Your selection will be shown on the left, the computer's on the right.
You will be told after each trial whether you WIN, LOSE or DRAW.
The computer will play in a certain way throughout the blocks.

Remember that:

ROCK beats SCISSORS
PAPER beats ROCK
SCISSORS beats PAPER

Press YELLOW button to start.

Summary of the one-way repeated measures ANOVA on arc-sine transformed proportion data for trial *n* item selection and outcome data

Metric	df	F	MSE	*p*	η_p^2
Item (I)	2,60	1.335	.034	.271	.043
Outcome (O)	2,60	1.387	.008	.258	.044

Note: Statistical significance in bold

Summary of the three-way repeated measures ANOVA on arc-sine transformed proportion data for first-order repetition effects

Metric	df	F	MSE	*p*	η_p^2
Item (I)	2,60	1.049	.002	.357	.034
Outcome (O)	2,60	0.049	.002	.952	.001
Strategy (S)	2,60	3.144	.978	.050	.095
I x O	4,120	2.236	.001	.069	.069
**I x S**	**4,120**	**2.830**	**.241**	**.028**	**.086**
**O x S**	**4,120**	**6.917**	**.299**	**<.001**	**.187**
I x O x S	8,240	1.814	.062	.075	.057

Note: Statistical significance in bold

Summary of the two-way repeated measures ANOVA on arc-sine transformed proportion data for second-order repetition effects

Metric	df	F	MSE	*p*	η_p^2
Strategy n (Sn)	2,60	0.737	.001	.483	.024
Strategy n+1 (Sn+1)	2,60	2.445	.316	.095	.075
**Sn x Sn+1**	**4,120**	**12.461**	**.057**	**<.001**	**.293**

Note: Statistical significance in bold

Pre-RPS agency manipulation (where computer guesses correctly)

Think of a number from 1 to 9 [space]

Take away 5 from the number you have [space]

Multiply that number by 9 [space]

If you have a two digit number, add those numbers together [space]

You should now have a single digit in mind [space]

Select the letter in the alphabet that matches your number (1 = A , 2 = B etc) [space]

Think of a European country beginning with that letter [space]

Think of an animal beginning with the last letter of that country [space]

Think of a fruit beginning with the last letter of the animal [space]

Were you thinking of Denmark, Kangaroo, Orange? [y / n]

Have you ever played this game before? [y / n]

Pre-RPS agency manipulation (where computer guesses incorrectly)

Think of a number from 1 to 9 [space]

Take away 5 from the number you have [space]

Multiply that number by 9 [space]

If you have a two digit number, add those numbers together [space]

You should now have a single digit in mind [space]

Select the letter in the alphabet that matches your number (1 = A , 2 = B etc) [space]

Think of a European country beginning with that letter [space]

Think of an animal beginning with the last letter of that country [space]

Think of a fruit beginning with the last letter of the animal [space]

Were you thinking of France, Elephant, Tomato? [y / n]

Have you ever played this game before? [y / n]
